# Pain Elimination during Injection with Newer Electronic Devices: A Comparative Evaluation in Children

**DOI:** 10.5005/jp-journals-10005-1240

**Published:** 2014-08-29

**Authors:** Neha Bansal, Sonali Saha, JN Jaiswal, Firoza Samadi

**Affiliations:** Postgraduate, Department of Pedodontics and Preventive Dentistry, Sardar Patel Post Graduate Institute of Dental and Medical Sciences Lucknow, Uttar Pradesh, India; Senior Lecturer, Department of Pedodontics and Preventive Dentistry, Sardar Patel Post Graduate Institute of Dental and Medical Sciences Lucknow, Uttar Pradesh, India; Professor and Director, Department of Pedodontics and Preventive Dentistry, Sardar Patel Post Graduate Institute of Dental and Medical Sciences Lucknow, Uttar Pradesh, India; Professor and Head, Department of Pedodontics and Preventive Dentistry, Sardar Patel Post Graduate Institute of Dental and Medical Sciences Lucknow, Uttar Pradesh, India

**Keywords:** Transcutaneous electrical nerve stimulation, Comfort control syringe, Conventional syringe, Anxiety

## Abstract

**Aim:** The present study was taken up to clinically evaluate and compare effectiveness of transcutaneous electrical nerve stimulator (TENS) and comfort control syringe (CCS) in various pediatric dental procedures as an alternative to the conventional method of local anesthesia (LA) administration.

**Materials and methods:** Ninety healthy children having at least one deciduous molar tooth indicated for extraction in either maxillary right or left quadrant in age group of 6 to 10 years were randomly divided into three equal groups having 30 subjects each. Group I: LA administration using conventional syringe, group II: LA administration using TENS along with the conventional syringe, group III: LA administration using CCS. After LA by the three techniques, pain, anxiety and heart rate were measured.

**Statistical analysis:** The observations, thus, obtained were subjected to statistical analysis using analysis of variance (ANOVA), student t-test and paired t-test.

**Results:** The mean pain score was maximum in group I followed by group II, while group III revealed the minimum pain, where LA was administered using CCS. Mean anxiety score was maximum in group I followed by group II, while group III revealed the minimum score. Mean heart rate was maximum in group I followed in descending order by groups II and III.

**Conclusion:** The study supports the belief that CCS could be a viable alternative in comparison to the other two methods of LA delivery in children.

**How to cite this article:** Bansal N, Saha S, Jaiswal JN, Samadi F. Pain Elimination during Injection with Newer Electronic Devices: A Comparative Evaluation in Children. Int J Clin Pediatr Dent 2014;7(2):71-76.

## INTRODUCTION

Anxiety and fear in children has continued to generate a lot of interest in pediatric dentistry. Although the purpose of anesthesia is to eliminate pain in a particular area, the actual method of delivering the anesthetic drug is anxiety provoking and painful because of stimulation produced by needle insertion and injection of anesthetic solution.^1^

In quest to search for techniques that minimize anxiety and pain, methods, such as transcutaneous electrical nerve stimulation (TENS) and comfort control syringe (CCS) with the conventional syringe technique, have been introduced.

The present study has been taken up to clinically evaluate and compare the effectiveness of TENS and CCS in various pediatric dental procedures as an alternative to the conventional method of local anesthesia (LA) administration.

## MATERIALS AND METHODS

The study was conducted on 90 healthy children who reported to department of pedodontics and preventive dentistry. The institutional review board and ethical committee approval was obtained for the study. Informed consent was obtained from the parents before starting any treatment or administration of drug. Subjects having at least one deciduous molar tooth indicated for extraction in either maxillary right or left quadrant in the age group of 6 to 10 years were selected for the study.

### Inclusion Criteria

- Children with no previous dental experience.
- Children mentally capable of communicating, satisfying the criteria of group I of the ASA guidelines as issued by the American Association of Anesthesiologists (1963).
- No history of allergy to any local anesthetic agent.
- No history of abscess in the tested area.
- No bleeding disorders.

### Exclusion Criteria

- Extremely anxious and fearful subjects.
- Abscess in the area of study.
- Presence of behavioral problems.
- Presence of perceptual motor problem.
- Immunocompromised patients.
- Systemic or genetic diseases that may compromise the health of oral mucosa.
- History of allergy to any of the components of agents to be used in the study.

**Fig. 1:**
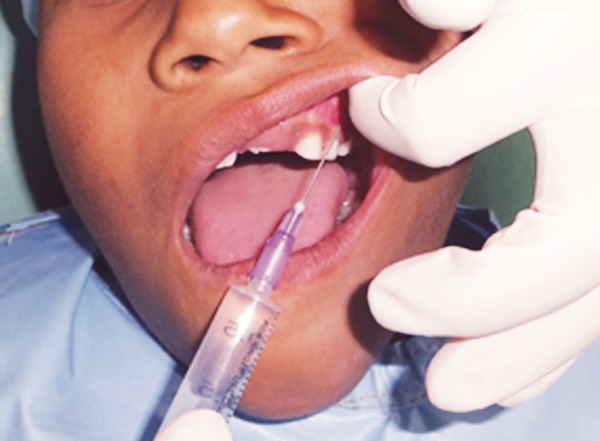
Local anesthesia administration using conventional syringe

**Fig. 2:**
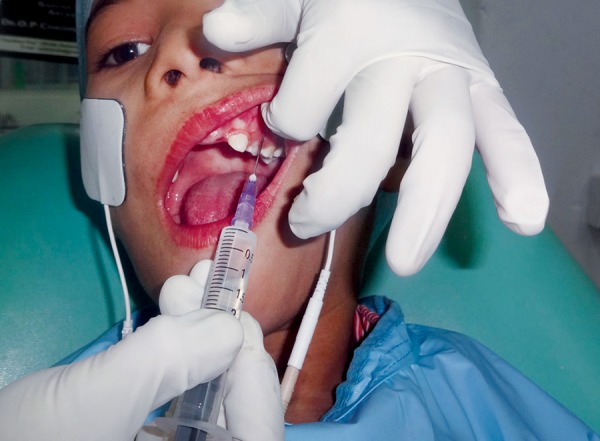
Local anesthesia administration using combination of TENS and conventional syringe

The child was made to sit on the dental chair comfortably and confidence was gained before the start of the procedure. The dialogue for all the three techniques was standardized as follows:

‘Your tooth will be made to sleep now with the help of a sleepy pen. I will put this pen next to your tooth and the sleepy juice will get out of this pen making your tooth go to sleep.’

Thus, information on the procedure of local anesthesia delivery was the same for all study subjects in the three groups. It was felt that any attempt to provide emotional support would introduce bias into the study.

Subjects were randomly divided into three study groups which were as follows:

*Group I:* Traditional LA administration using conventional syringe (Fig. 1).

Comprised of 30 subjects in whom LA was administered using conventional syringe and needle.

*Group II:* Local anesthesia administration using TENS along with the conventional syringe (Fig. 2).

Comprised of 30 subjects in whom LA was administered using TENS in combination with conventional syringe. The apparatus used was transcutaneous nerve stimulator marketed by Skylark Device and Systems Co, Ltd, Taiwan. This TENS system consists of two skin electrodes and a hand held digital meter. This meter has a knob which controls the current level and can be turned by the patient himself. Thus, the patient can control the level of anesthesia.

The site of electrode pad placement was gently swabbed with surgical spirit to remove any skin oils or substances interfering with current  fow. For maxillary primary molars, electrode pads were placed over the apices of the primary molars just below zygoma.

**Fig. 3:**
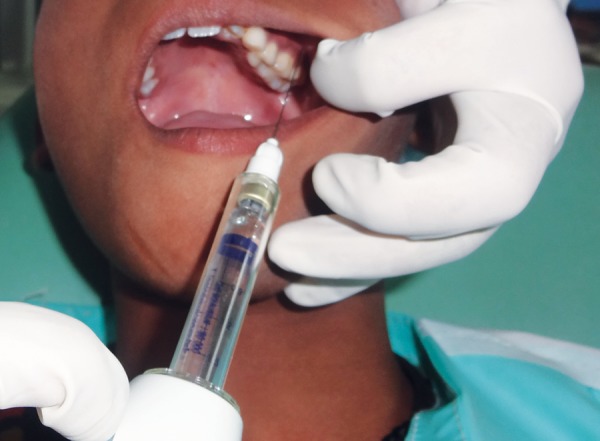
Local anesthesia administration using comfort control syringe

Since, it is a combination procedure, LA was then administered using conventional syringe as described in group I. After LA administraion by TENS system and conventional syringe method, again the response of the child was noted using the above-mentioned criteria, including pain score, which was evaluated using Wong-Baker’s scale.^2^

*Group III:* Local anesthesia administration using CCS (Fig. 3).

Thirty subjects were taken in this group, and LA was administered using CCS.

The apparatus used was Midwest Comfort Control^TM^ Syringe marketed by Denstply Professional, USA, consisting of a control unit, hand piece, needle (27 gauge) and disposable anesthetic cartridge and syringe sleeves.

**Fig. 4:**
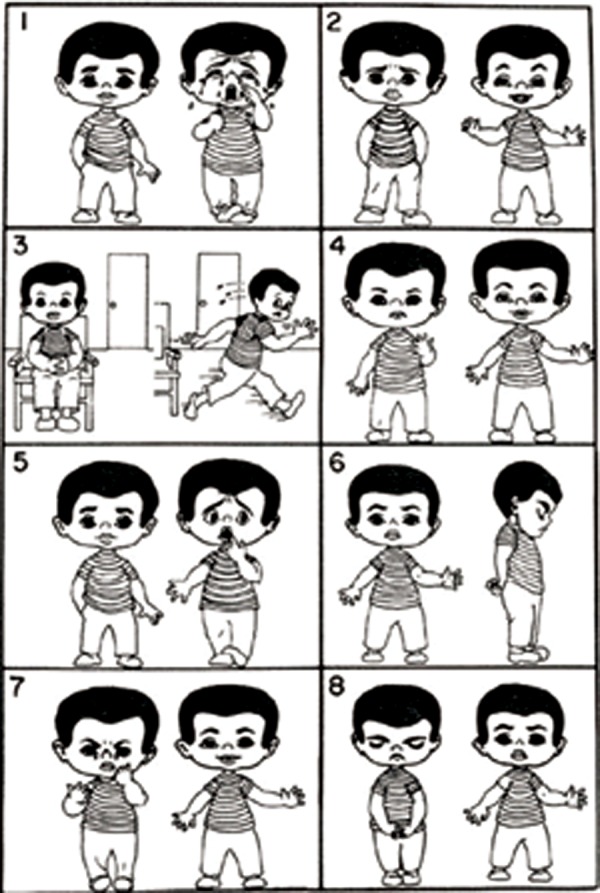
Venham’s picture scale

**Fig. 5:**
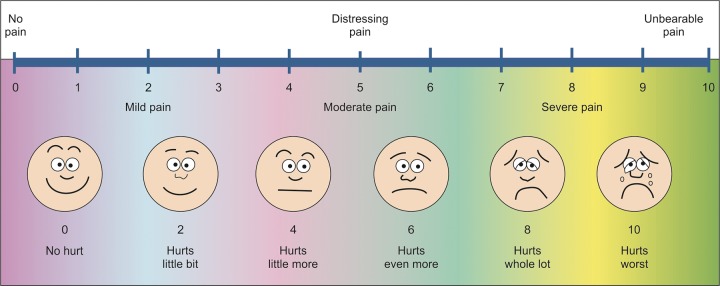
Wong-Baker’s facial pain scale

A standardized procedure was used for all the three groups:

- No topical anesthesia used.
- All injections consisted of 2% lidocaine with 1:200,000 epinephrine.
- Only buccal (0.90 ml) and palatal infltrations (0.45 ml) were given.
- Injection of the local anesthetic agent was given slowly with average duration of nearly 2 minutes for approximately 1 ml per minute.
- Before and after administration of LA, response of the child was noted using the following criteria:
  – Anxiety score (using Venham’s picture scale)^3^ (Fig. 4).
  – Heart rate (using pulse oximeter).
- Pain score was evaluated using Wong-Baker’s scale after administration of LA in all the three groups (Fig. 5).

Thereafter, extraction was performed in all groups using extraction forceps.

## STATISTICAL ANALYSIS

The observations, thus, obtained were subjected to statistical analysis using analysis of variance (ANOVA), student t-test and paired t-test were performed to know the effect of each variable and to reveal the statistical significance. The confidence level of study was proposed to be 95%; hence, p-value < 0.05 has been considered significant, p-value < 0.01 has been considered highly significant and p-value <0.001 has been considered very highly significant.

## RESULTS

Table 1 shows mean anxiety scores before and after intervention between groups I, II and III.

Mean anxiety score was maximum in group I followed by group II, while group III revealed the minimum score.

Table 2 shows the mean heart rate in the three study groups. The mean heart rate was maximum in group I followed in descending order by groups II and III.

Table 3 shows the mean pain score in the three study groups.

The mean pain score was maximum in group I followed by group II, while group III revealed the minimum pain where LA was administered using CCS.

## DISCUSSION

Pain control is an indispensable part of modern dentistry. Prevention of pain during dental procedures can nurture relationship of the patient and dentist, building trust, allaying fear and anxiety, thus promoting a positive dental attitude. Pain control measure, such as administration of LA, the most common modality of pain control in dental procedures, in itself, is a source of fear and anxiety for pediatric dental patients.^4^

**Table 1:** **Table 1:** Change in anxiety scores in different study groups

*S. no.*		*Groups*		*Before intervention * *(n = 30)*				*After intervention* * (n = 30)*				*Change in anxiety score*				*Significance of change* * (paired t-test)*			
				*Mean*		*SD*		*Mean*		*SD*		*Mean*		*SD*		*t-value*		*p-value*	
1.		I		6.27		0.91		6.73		0.98		0.47		0.97		2.626		0.014	
2.		II		6.00		0.87		6.03		0.93		0.03		1.00		0.183		0.856	
3.		III		5.17		1.34		4.03		1.13		–1.13		1.81		3.421		0.002	

**Table 2:** **Table 2:** Change in heart rate in different study groups

*S. no.*		*Groups*		*Before intervention* *(n = 30)*				*After intervention* *(n = 30)*				*Change in heart rate*				*Significance of change* *(paired t-test)*			
				*Mean*		*SD*		*Mean*		*SD*		*Mean*		*SD*		*t-value*		*p-value*	
1.		I		106.6		3.1		110.4		7.0		3.8		6.6		3.169		0.004	
2.		II		105.2		3.9		108.5		5.0		3.3		4.4		4.090		<0.001	
3.		III		103.9		3.2		88.2		5.4		–15.7		4.6		18.700		<0.001	

**Table 3:** **Table 3:** Comparative evaluation of pain of children in different groups after administration of local anesthetic drug

*S. no.*		*Pain score* *(Wong-Baker’s scale)*		*Group I (n = 30)*				*Group II (n = 30)*				*Group III (n = 30)*			
				*No.*		*%*		*No.*		*%*		*No.*		*%*	
1.		0		0		0.0		0		0.0		2		6.7	
2.		2		0		0.0		1		3.3		19		63.3	
3.		4		7		23.3		8		26.7		7		23.3	
4.		6		12		40.0		17		56.7		2		6.7	
5.		8		10		33.3		4		13.3		0		0.0	
6.		10		1		3.3		0		0.0		0		0.0	
Mean ± SD					6.33 ± 1.67				5.60 ± 1.43				2.60 ± 1.40		

F = 51.819; p < 0.001 (ANOVA)

To overcome the drawbacks of LA, alternative modes of pain management, such as use of TENS along with the conventional syringe and CCS, are available to us.

In the present study, comparative evaluation of anxiety score of children in different groups before and after LA administration revealed that anxiety in children of group I (conventional syringe) was significantly higher followed in descending order by group II (TENS and conventional syringe) and least in group III CCS.

In accordance to the results of the present study, Nicholson et al,^4^ Sculean et al^5^ and Langthasa et al^6^ concluded that the computer-controlled LA delivery system was more acceptable and less anxiety provoking as compared to the conventional syringe method of LA delivery.

Oztas et al (2006) stated that computer-controlled method of LA delivery provided a virtually pain-free, predictable injection and had the potential of desensitizing patients to their fears of injections, thus, decreasing anxiety.^7^

This may be attributed to its pen-like design which is less threatening to the patients and does not look like a syringe indicating that physical appearance of a dental injector is of importance to children. Additionally, more comfortable placement of the needle was possible because dentist could hold this system within inches of the needle and use a  finger pen grasp as used with other dental instruments. Thus, computer-controlled method of LA delivery could be an useful alternative to the conventional method alleviating anxiety in pediatric dental patients as suggested by Grace et al.^8^

Results of the present study were in accordance to the study conducted by Dhinsa^9^ etal and Wilson et a^10^ who suggested TENS to be a less anxiety provoking technique and well accepted by patients. Improvements in the clinical behavior for the categories of movement and crying during injection was found when TENS was used as compared to conventional syringe method.

The anxiety status in children of group II (combination of TENS with conventional syringe) was more as compared to group III CCS, before and after the administration of LA which according to Malamed et al (1989) may be attributed to the fact that the TENS depended upon the ability of the patients to understand the concept involved, i.e. the mature patients could successfully perceive and receive TENS as compared to young pediatric patients.^11^

Thus, the results of the present study indicated that the CCS method of LA administration was the most acceptable and least anxiety provoking among all the three study groups.

### Pain Perception

In the present study, pain perception in group I (conventional syringe) was more as compared to that in group II (combination of TENS and conventional syringe) during the administration of LA.

Studies by Te Duits et al (1998) and Munshi et al (2000) inferred that pain perception of patient was significantly reduced with use of TENS and preferred by children when compared with LA by conventional syringe method.^12,13^

On the contrary, Jones et al (1996), Cho et al (1998) and Lodaya et al (2010) suggested TENS to be an useful adjunct in providing pain control during dental care in children.^14^

Pain perception in children when compared between in groups I (conventional syringe) and III CCS showed the pain to be more in group I compared to III, during the administration of LA.

Study results as inferred by Nicholson et al (2001), Sculean et al (2004) and Langthasa et al (2012) states that CCS delivers a controlled pressure and volume, minimizing tissue distension, eliminating the need for heavy finger pressure and preventing too rapid injection. The pen grasp permitted needle rotation as it penetrates the tissue to minimize needle defection resulting from tissue refection.^4-6^

According to Pashley et al (1981), painful sensation during any needle injection comes from administering anesthetic solution too rapidly or with much force. Injection pressures also vary widely because of the wide variance in soft tissue elasticity. He also stated that with conventional manual syringe, the volume fow and pressure parameters cannot be precisely controlled resulting in difficult, erratic and uncomfortable injections.^14,15^

According to Krochak et al (1998), this system also maintains a constant positive pressure on the  fow of anesthetic solution, thereby yielding a virtually pain-free needle insertion.^16^

Another reason for less painful injections by CCS could be the improved tactile sensation. Minimal force was required during administration, maintaining optimal fow rate resulting in a reduction of pain during injection as attributed by Hochman et al.^17^ So, the improved tactile feedback, visibility and automated aspiration allows concentration on needle positioning and patient interaction.

When comparing pain ratings in children of groups II (combination of TENS and conventional syringe) and III CCS, the results of the present study showed that pain was more in group II as compared to group III, during the administration of LA.

No studies till date have compared both these techniques of local anesthesia delivery. As inferred by the present study, computer control LA delivery system is superior in reduction of pain when compared with the TENS. This may be attributed to the design of the hand piece and also the precise and controlled delivery of LA in this particular system.

Thus, result, the present study stated that CCS method of LA administration was the least painful among all the three study groups.

### Heart rate

Comparing heart rate between the three study groups, results showed that heart rate was maximum in group I (conventional syringe group), before and after administration of LA followed in descending order by group II (combination of TENS and conventional syringe) and least in group III (CCS group).

Changes in the heart rate are expected to refect patient responsiveness to procedures, especially during stressful experiences. Salient stimuli like pain may result in increased heart rate which is the primary mode of cardiovascular response in young children to perceive stressful conditions. According to Dowling^18^ et al, heart rate increased in response to the application of pain.

No studies have yet been conducted evaluating heart rate before and after needle prick using TENS compared to the conventional syringe method of LA delivery. Decreased heart rate in group II (combination of TENS) may be due to the patient’s relaxation and reduction of fear when they realized that anesthesia was adequate and the procedure was not painful leading to decrease in stress associated tachycardia.

In accordance to the results of the present study, Myers et al (1972), Cho et al (1998) and Wilson et al (1999) also inferred significant difference in the mean heart rate and percent change in the heart rate when electronic dental anesthesia was used alone.^10,19,20^

According to Lopez et al (2005) inferred that lower heart rate using computerized controlled delivery system indicated less painful injections.^21,22^

Results of the present study indicate that heart rate was least when LA was administered using CCS. This may be attributed to the fact that the injection administered by CCS technique was virtually pain free, more predictable and more importantly its pen-like design was less threatening to the patients.

Since, results indicated least heart rate when CCS was used, it would be justified to state that CCS was the most acceptable mode of LA administration to the pediatric patient when evaluated in respect to anxiety, pain perception and heart rate.

## CONCLUSION

On the basis of the results, observations and statistical analysis, the following conclusions were drawn:

1. Children showed most anxious behavior when local anesthesia was administered using conventional syringe followed in descending order by electronic dental anesthesia in combination with conventional syringe and least by the CCS alone.
2. Comfort control syringe was the most accepted and least stress inducing method of LA administration.
3. Comfort control syringe was found to be least painful during administration of LA during needle prick as compared to electronic dental anesthesia (in combination with conventional syringe) and conventional syringe alone.
4. Minimum changes in the heart rate were observed when LA was administered using CCS.

Thus, it was concluded from the study that CCS could be a viable alternative in comparison to the other two methods of LA delivery in children.
